# Injection-Site Abscess Caused by Mycobacterium abscessus Following Testosterone Injection: A Case Report

**DOI:** 10.7759/cureus.109748

**Published:** 2026-05-27

**Authors:** Khaled Abubaker, Hamzeh Alshahwan, Omar Kreeshan, Marie Kima

**Affiliations:** 1 Internal Medicine, HCA Florida North Florida Hospital, Gainesville, USA; 2 Infectious Diseases, HCA Florida North Florida Hospital, Gainesville, USA

**Keywords:** anti-bacterial agents, immuno-competent, infected wound, nontuberculous mycobacteria (ntm), skin and soft tissue

## Abstract

*Mycobacterium abscessus* is a rapidly growing nontuberculous mycobacterium that is increasingly recognized as a cause of skin and soft tissue infections, particularly following injection procedures, cosmetic interventions, and surgical wounds. Although traditionally associated with immunocompromised hosts, a growing body of evidence demonstrates that *M. abscessus* can cause significant disease in immunocompetent individuals. We describe a 72-year-old immunocompetent male who developed a persistent left thigh abscess following a routine testosterone injection. Despite initial empiric antibiotic therapy, wound cultures ultimately grew *M. abscessus*, necessitating prolonged multidrug antimycobacterial therapy. The hospital course was further complicated by acute respiratory failure requiring intensive care admission and mechanical ventilation. This case underscores the importance of maintaining a high index of suspicion for atypical mycobacterial pathogens in non-resolving injection-site infections, even in patients without apparent immunodeficiency, and highlights the challenges associated with diagnosis and prolonged treatment of *M. abscessus* soft tissue infections.

## Introduction

Rapidly growing nontuberculous mycobacteria (NTM), including *Mycobacterium abscessus*, are environmental organisms ubiquitously found in soil, dust, and water sources [1]. *M. abscessus* is often regarded as one of the most antibiotic-resistant mycobacteria and is the second most frequent NTM pathogen causing human disease [2]. The *M. abscessus* complex (MABC) comprises three subspecies - abscessus, massiliense, and bolletii - each with distinct antimicrobial resistance profiles that have important therapeutic implications [3,4].

While pulmonary disease remains the most common manifestation of *M. abscessus* infection, skin and soft tissue infections (SSTIs) represent a significant and increasingly reported clinical entity [1,5]. These infections are predominantly associated with surgical or injection procedures, cosmetic interventions, tattooing, and contaminated medical devices [6,7]. Notably, *M. abscessus* accounts for the greatest number of postinjection or iatrogenic infections among atypical mycobacteria [7]. The organism's ability to form dense biofilms on surfaces and its resistance to common disinfectants allow it to persist in water distribution systems and on medical equipment, serving as a constant reservoir for infection [2,8].

Several outbreaks of *M. abscessus* postinjection abscesses have been documented worldwide, linked to contaminated injectable preparations, improper sterilization of equipment, and reuse of multi-dose vials [9-12]. In the largest reported U.S. outbreak, 87 persons developed postinjection abscesses from a contaminated injectable medication, with all cultures yielding identical *M. abscessus* strains [12]. Similar outbreaks have been reported following corticosteroid injections, lipodissolve procedures, and other intramuscular injections [7,13]. Intramuscular testosterone injections have been implicated in cases of pyomyositis and soft tissue infection, though *M. abscessus* as the causative organism in this context remains exceedingly rare [14].

Extrapulmonary *M. abscessus* infections predominantly affect immunocompetent patients (62%), with healthcare-associated exposures accounting for 44% of cases [15]. Despite this, diagnosis is frequently delayed due to the organism's slow growth on standard culture media and the low clinical suspicion for atypical mycobacteria in routine injection-site infections [5,6]. Treatment is notoriously challenging, requiring prolonged combination antimicrobial therapy often exceeding six months, frequently combined with surgical debridement [3,15]. We present a case of *M. abscessus* soft tissue infection in an adult without apparent immunodeficiency following a testosterone injection, highlighting the diagnostic challenges and complex management required for this emerging pathogen.

## Case presentation

A 72-year-old man with hypertension and prediabetes presented with one week of progressive swelling, erythema, and severe pain in the left thigh beginning the day after a testosterone injection in an outpatient clinic (Figure 1). He also reported fevers up to 102°F. His medical history was otherwise unremarkable, with no history of HIV infection, malignancy, organ transplantation, or use of corticosteroids or other immunosuppressive medications. On arrival, he was febrile but hemodynamically stable. Laboratory evaluation was notable only for a creatinine of 1.39 mg/dL with no evidence of neutropenia or other hematologic abnormalities.

**Figure 1:**
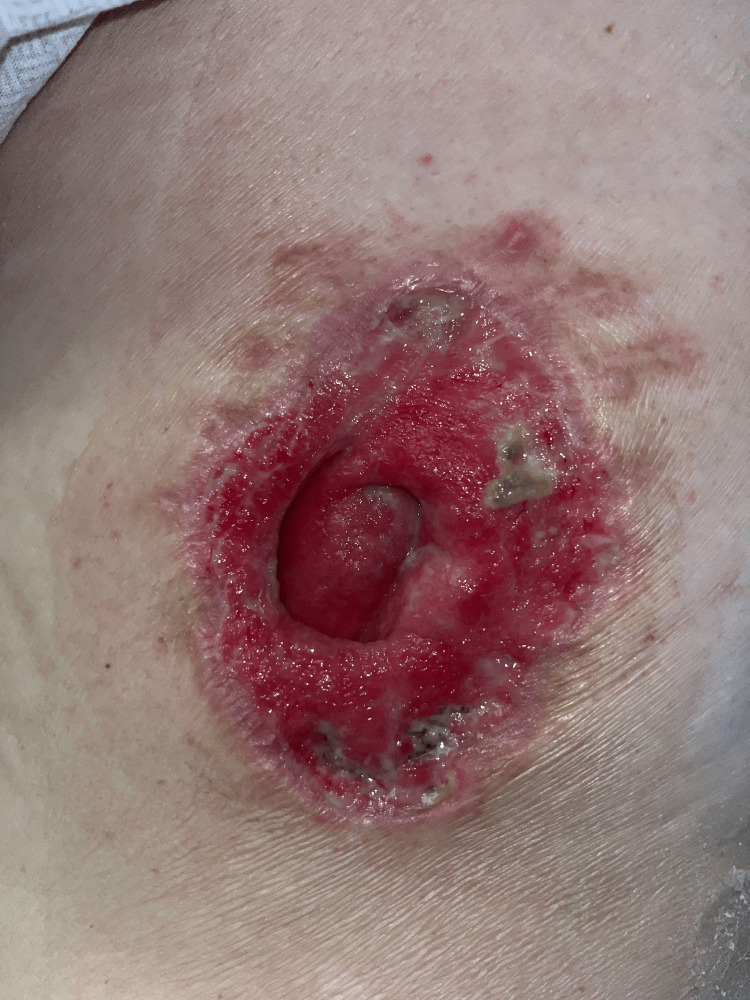
Left thigh showing diffuse erythema, swelling, and induration at the site of intramuscular testosterone injection, consistent with an injection site abscess.

Axial contrast-enhanced CT of the left thigh revealed a 3.7 cm low-density intramuscular collection within the rectus femoris with surrounding soft tissue edema (Figure 2). Subsequent MRI of the left thigh demonstrated an early organizing collection measuring 4.4 × 2.6 cm without evidence of gas formation (Figure 3).

**Figure 2:**
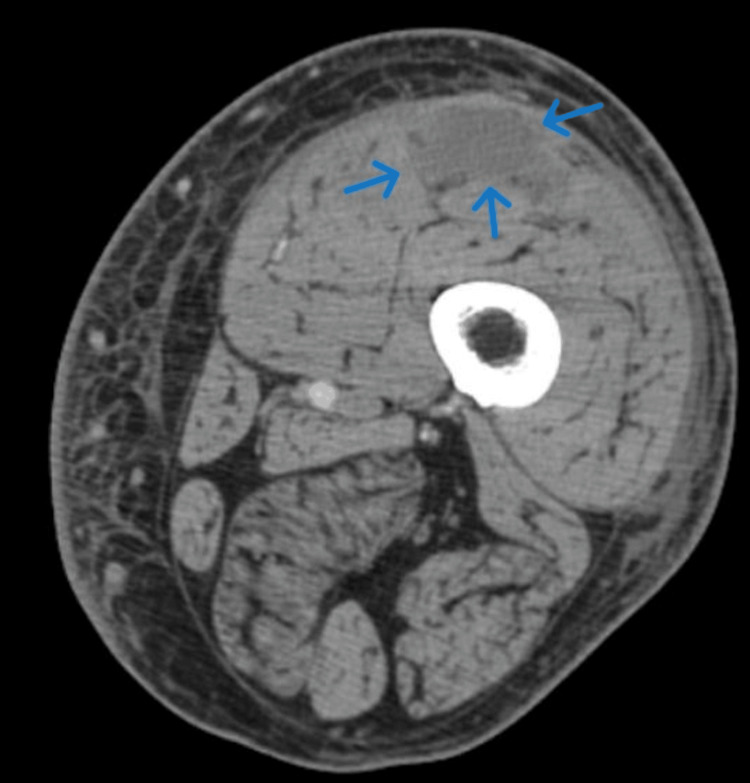
Axial contrast-enhanced computed tomography (CT) of the left thigh demonstrating a 3.7 cm low-density intramuscular fluid collection within the rectus femoris muscle (blue arrows), with surrounding soft tissue edema and inflammatory stranding of the adjacent musculature, consistent with an intramuscular abscess.

**Figure 3:**
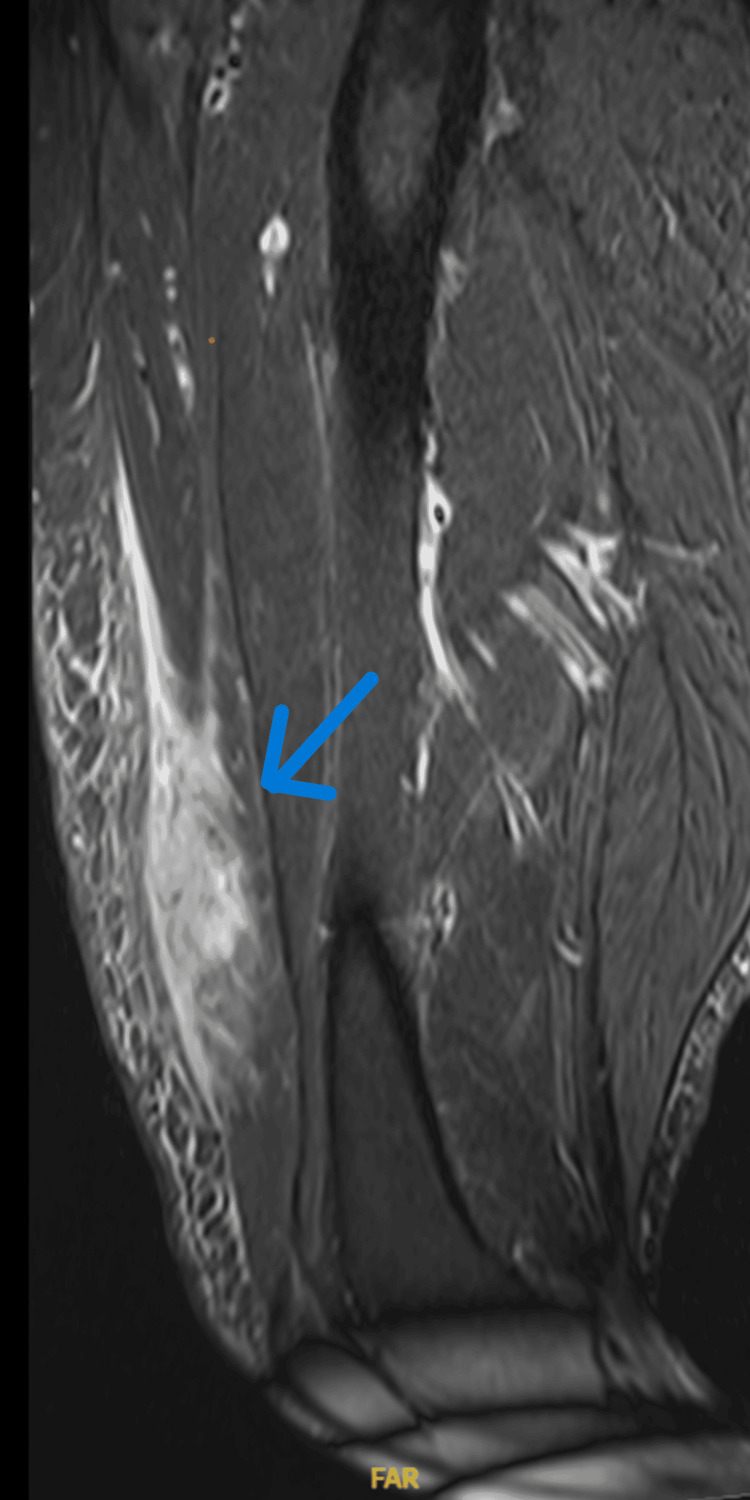
Magnetic resonance imaging (MRI) of the left thigh demonstrating an early organizing intramuscular collection measuring 4.4 × 2.6 cm within the rectus femoris muscle (blue arrow), without evidence of gas formation or extension into adjacent compartments, consistent with an evolving intramuscular abscess.

Empiric cefepime and vancomycin were initiated. Blood cultures remained negative, but wound cultures grew Gram-positive rods initially. Thus, he underwent incision and drainage by an orthopedist. Intraoperative cultures subsequently identified *Mycobacterium*, and antimicrobial therapy was held pending final speciation.

*M. abscessus*was isolated after five days. Antimicrobial therapy was started targeting it with azithromycin, amikacin, and cefoxitin. During a brief interruption in wound-vac therapy, the patient developed another recurrent collection requiring additional debridement. Intraoperative cultures grew *Staphylococcus epidermidis*. Thus, daptomycin was added. Amikacin was held due to worsening kidney function.

His hospital course was complicated by gradual onset of worsening hypoxia, requiring ICU transfer, intubation, and mechanical ventilation. Chest radiography revealed bilateral lower lung zone infiltrates, and CT of the chest demonstrated bilateral nonspecific alveolar infiltrates with air bronchograms and small bilateral pleural effusions. The differential diagnosis included daptomycin-induced pneumonitis versus hospital-acquired pneumonia. Bronchoalveolar lavage (BAL) was performed, which showed a normal eosinophil count, effectively ruling out daptomycin-induced pneumonitis. BAL cultures and Gram stain showed no growth, and a viral respiratory panel was negative. A clinical diagnosis of hospital-acquired pneumonia was made, and the patient was treated empirically with vancomycin and levofloxacin. Daptomycin was discontinued, and antimycobacterial therapy was temporarily held.

Once stabilized, azithromycin and cefoxitin were restarted, and amikacin was resumed after renal function improved. The organism was identified as *M. abscessus* by 16S rRNA gene sequencing. Drug susceptibility testing was conducted using broth microdilution per Clinical and Laboratory Standards Institute (CLSI) guidelines, with extended 14-day incubation for clarithromycin to assess inducible macrolide resistance, demonstrating intermediate susceptibility to imipenem and cefoxitin. Subspecies-level identification and erm(41) gene testing were not performed. Based on these results, the regimen was transitioned to linezolid, azithromycin, and intravenous amikacin. He required a tracheostomy for prolonged ventilatory support and was ultimately discharged to a long-term acute care facility on this triple therapy for a planned one-month intensive course. A summary of the antimicrobial timeline is provided in Table 1.

**Table 1:** Chronological summary of antimicrobial therapy during hospitalization for injection-site M. abscessus abscess HAP: hospital-acquired pneumonia; LTAC: long-term acute care; IV: intravenous; *M. abscessus*: *Mycobacterium abscessus*; *S. epidermidis*: *Staphylococcus epidermidis*; BAL: bronchoalveolar lavage

Phase	Timeframe	Antimicrobial Regimen	Rationale
Empiric therapy	Hospital day 1	Cefepime + vancomycin	Empiric coverage pending cultures
Culture-directed (initial)	Upon mycobacterial identification	Azithromycin + amikacin + cefoxitin	Guideline-based initial intensive phase for *M. abscessus*
Modification 1	During wound-vac interruption	Azithromycin + cefoxitin + daptomycin (amikacin held)	*S. epidermidis* co-infection; amikacin held for worsening renal function tests
HAP treatment/antimycobacterial hold	ICU transfer	Vancomycin + levofloxacin; daptomycin discontinued; antimycobacterial therapy held	Clinical diagnosis of HAP; daptomycin-induced pneumonitis excluded by BAL
Resumption	Post-stabilization	Azithromycin + cefoxitin + amikacin (after renal recovery)	Resumption of intensive phase
Final regimen/discharge	Discharge to LTAC	Linezolid + azithromycin + IV amikacin (planned one-month course)	Based on final susceptibility results

## Discussion

This case demonstrates that *M. abscessus* can cause significant SSTI even in adults without apparent immunodeficiency, particularly following intramuscular injections. In a large French retrospective study of 45 patients with extrapulmonary *M. abscessus* infections, 62% had no reported immunodeficiency, and healthcare-associated exposures accounted for 44% of cases [15]. Similarly, a U.S. epidemiological study of extrapulmonary NTM infections found that rapidly growing NTM, including *M. abscessus*, were most prevalent in southern states, with skin and soft tissue being the most common extrapulmonary site of infection [16].

The pathogenesis of *M. abscessus* injection-site infections is closely linked to the organism's environmental ubiquity and its remarkable ability to form biofilms. *M. abscessus* can adhere to surfaces and develop dense biofilms more rapidly than other rapidly growing mycobacteria, which contributes to its enhanced pathogenicity and resistance to antimicrobial treatment [2,8]. This biofilm-forming capacity, combined with resistance to common disinfectants, allows the organism to persist in water distribution systems and on medical equipment [1,17]. Multiple outbreaks have been traced to contaminated injectable preparations, improper sterilization practices, and reuse of multi-dose vials with contaminated diluents [9,11-13]. In our patient, the testosterone injection administered in an outpatient clinic likely served as the portal of entry, consistent with the well-documented association between intramuscular injections and *M. abscessus* soft tissue infections [10,14].

Diagnosis of *M. abscessus* SSTIs is frequently delayed, with a median time to diagnosis of 69 days reported in one surveillance study [18]. The classic presenting signs include purulent drainage, violaceous nodules, and subcutaneous abscesses at the site of a recent procedure, with persistence of infection despite standard debridement and empiric antibiotic treatment being a key diagnostic clue [6]. A high index of clinical suspicion is essential, as standard bacterial cultures may fail to identify the organism, and mycobacterial-specific growth media are required for definitive diagnosis [5,19]. In our patient, the initial wound cultures grew Gram-positive rods, which may have contributed to diagnostic delay, as the morphology was not immediately recognized as suggestive of mycobacterial infection. Definitive identification of *M. abscessus* was not achieved until day 5 of culture, underscoring the importance of maintaining mycobacterial cultures even when initial Gram stain results suggest a more common pathogen.

*M. abscessus* is intrinsically resistant to many conventional antibiotics due to multiple resistance mechanisms involving efflux pumps, inactivating enzymes, and low drug-target affinity [2,20]. Subspecies identification is critical for guiding therapy, as subspecies *abscessus *and *bolletii *harbor a functional erm(41) gene conferring inducible macrolide resistance, whereas subspecies *massiliense* lacks this functional gene and remains macrolide-susceptible [4,21]. In our case, subspecies-level identification and erm(41) gene testing were not performed, which represents a limitation in tailoring macrolide-based therapy. This highlights the challenges encountered in resource-limited settings where advanced molecular diagnostics may not be readily available. The current recommended initial treatment regimen includes a macrolide (clarithromycin or azithromycin), amikacin, and a parenteral agent such as cefoxitin or imipenem during an intensive phase, followed by a continuation phase with oral agents [3,22]. In the largest reported outbreak of postinjection *M. abscessus* abscesses, combination therapy with surgical excision and clarithromycin for three to six months achieved a 95% success rate, compared with less than one-third success with either modality alone [9].

Management of extrapulmonary *M. abscessus* infections requires a combination of surgical debridement and prolonged multidrug therapy tailored to susceptibility testing. In the French cohort study, cure was achieved in 80% of patients who received a median antimicrobial regimen of six months, with 55% also undergoing surgical intervention [15]. Treatment duration for extrapulmonary infections typically requires at least six months, though some experts advocate for longer courses depending on clinical response and subspecies identification [15,21]. Our patient's course illustrates several of these management challenges. Amikacin-related nephrotoxicity necessitated temporary interruption of a key antimicrobial agent, requiring careful balancing of treatment efficacy against drug toxicity. Additionally, recurrent abscess formation during a brief interruption in wound-vac therapy necessitated repeated surgical debridement, reinforcing the critical role of aggressive source control in achieving favorable outcomes. His hospital course was further complicated by acute respiratory failure and hospital-acquired pneumonia, consistent with the recognized morbidity associated with *M. abscessus* infections, which can prolong hospitalization and necessitate intensive care management.

This case adds to the limited but growing literature on *M. abscessus* soft tissue infections following intramuscular testosterone injections. While testosterone injection-associated pyomyositis has been previously reported, *M. abscessus* as the causative organism in this specific clinical context is exceedingly rare [14]. This case reinforces the need for clinicians to consider atypical mycobacterial pathogens in patients presenting with non-resolving injection-site infections, particularly when standard empiric therapy fails to produce clinical improvement.

## Conclusions

*M. abscessus* should be considered in cases of persistent or recurrent soft-tissue abscesses following injections, even in immunocompetent patients. Early recognition, appropriate culture techniques, prompt surgical management, and targeted multidrug therapy are essential for successful outcomes. Clinicians should maintain a high index of suspicion for atypical pathogens in patients presenting with non-resolving injection site infections, particularly in the context of cosmetic or nonstandard medical procedures.
